# Self-perception of health and quality of life in patients on renal replacement therapy undergoing hemodialysis: a case-control study

**DOI:** 10.1590/1516-3180.2024.0144.R1.14042025

**Published:** 2025-07-14

**Authors:** Diego José Gambin, Felipe Gomes Dallepiane, Leonardo Saraiva, Marciele Cristiane Spanenberg Führ, Fabiana Piovesan, Caroline Mirek, João Paulo De Carli, Micheline Sandini Trentin

**Affiliations:** I Faculdade de Odontologia, Universidade de Passo Fundo, Passo Fundo (RS), Brazil.; II Faculdade de Odontologia, Universidade Federal de Santa Catarina, Florianópolis (SC), Brazil.; III Faculdade de Odontologia, Universidade de Passo Fundo, Passo Fundo (RS), Brazil.; IV Faculdade de Odontologia, Universidade de Passo Fundo, Passo Fundo (RS), Brazil.; V Faculdade de Medicina, Universidade de Passo Fundo, Passo Fundo (RS), Brazil.; VI Faculdade de Odontologia, Universidade de Passo Fundo, Passo Fundo (RS), Brazil.; VII Faculdade de Odontologia, Universidade de Passo Fundo, Passo Fundo (RS), Brazil.; VIII Faculdade de Odontologia, Universidade de Passo Fundo, Passo Fundo (RS), Brazil.

**Keywords:** Kidney failure, chronic, Renal insufficiency, Quality of life, Systemic diseases, Medicine, Dentistry

## Abstract

**BACKGROUND:**

Chronic kidney disease (CKD) presents challenges to human health and quality of life, with care primarily focusing on renal function and comorbidity management. Several studies confirm the relationship between oral and systemic conditions of patients. Therefore, CKD and periodontal disease can be related because they are both inflammatory conditions that further increase the risk of other pathologies. The impact of CKD on oral health and overall quality of life is an area of interest.

**OBJECTIVES:**

To evaluate patients with CKD undergoing renal replacement therapy regarding the levels of self-perception of oral health and quality of life.

**DESIGN SETTING:**

This case-control study was conducted jointly at the Universidade de Passo Fundo and Hospital São Vicente de Paulo, Brazil.

**METHODS:**

This study included two patient groups: 1) Case group with CKD (CGA) comprising 116 patients; 2) Control group without CKD (CGO) composed of 124 patients. We used a structured questionnaire and the Oral Health Impact Profile (OHIP-14). We used the Chi-square and Wilcoxon-Mann-Whitney tests as well as an analysis of variance measure (P < 0.05).

**RESULTS:**

The systemic diseases most prevalent among our patient cohort included hypertension (16.9% CGO and 75.9% CGA) and diabetes mellitus (8.9% CGO and 38.8% CGA). The systemic health perception was good in 66.9% of the CGO group and average in 42.2% of CGA group members. Oral health perception was good in 46% of CGO and 50% of CGA group members. Results of the self-assessment for quality of life showed a statistically significant difference between the groups for physical domain, physical disability, and social disadvantage. A comparison between the control and case (CKD) groups, based on the OHIP-14 score, showed statistically significant differences in the functionality (P < 0.006), physical disability (P < 0.042), and social disadvantage (P < 0.031) domains for the CKD group.

**CONCLUSION:**

Patients with CKD have lower rates of self-perception of oral health and quality of life than individuals without CKD.

## INTRODUCTION

The prevalence of chronic kidney disease (CKD) is rapidly increasing worldwide, representing a significant financial burden for healthcare systems.^1^ CKD is classified into the following five stages: renal disorders with a regular glomerular filtration rate; renal disorders with a slight reduction in the glomerular filtration rate;^2^ moderate reduction in glomerular filtration rate;^3^ severe reduction in the glomerular filtration rate;^4^ and kidney failure.^5^ The glomerular filtration rate value reveals the level of kidney dysfunction to the clinician. 

 It is estimated that CKD has a worldwide prevalence of 11–13% among adults.^2^ However, CKD prevalence has increased both in the industrialized countries and worldwide, and this is important because of its high morbidity and mortality.^3-8^ Between 30% and 50% of all cases of end-stage renal failure may be attributed to diabetes mellitus and arterial hypertension.^9^ Nutritional factors and depression may impact the quality of life of individuals undergoing renal replacement therapy.^10,11^

Several studies have validated the relationship between the oral and systemic conditions of patients. Therefore, CKD and periodontal disease may be related because they are both inflammatory conditions that further increase the risk of other pathologies, such as diabetes, hypertension, atherosclerosis, liver diseases, and changes in the intestinal microbiota, thereby reducing the quality of life of individuals. Thus, the lack of oral health care in patients with CKD causes more serious clinical problems, affecting the quality of life and risk of transplant, and may even lead to death.^8,9^

However, several studies have shown an association between oral diseases and CKD, and regular oral care and instruction can be a relevant strategy to reduce CKD.^12-16^ Strategies for facing this global health problem include identifying risk factors associated with comorbidities and complications and adequately managing the pertinent issues.^13,14^

 Access to specialized public health service by the entire population, irrespective of their social condition or place of origin, is a premise and obligation of the SUS. This has not been previously evaluated in relation to ACS in Rio Grande do Norte. Given the group of highly complex procedures and the absence of well-defined flowcharts for access to the reference service, spatial analysis can aggregate informa tion for health managers and the scientific community. Additionally, the analysis of this sample establishes an epidemiological profile that characterizes an SUS referral service and provides an estimate of the prevalence of severe heart disease in a state in Northeastern Brazil. 

## OBJECTIVE

 This case-control study aimed to evaluate self-perception of the level of oral health and quality of life in patients with CKD undergoing renal replacement therapy by comparing the case group (having CKD) with the control group. 

## METHODS

 The present study was approved by the Research Ethics Committee of the Universidade de Passo Fundo, Brazil, in March 2020 (Ethical Approval Protocol Number 3.929.915), and by the Research Ethics Committee of the Hospital São Vicente de Paulo (HSVP) (No. 3.929.915), in Passo Fundo, Rio Grande do Sul, in November 2019. The methodology of this study was based on the Strengthening of the Reporting of Observational Studies in Epidemiology guidelines. 

### Study design

 This is a case-control observational study. The predictor variable is CKD, while the outcome variables are self-perception of oral health and quality of life. The participants were divided into two groups. The case group (Group 1; CGA; n = 116) included patients with CKD undergoing hemodialysis, and the control group (Group 2; CGO; n = 124) included patients without CKD. These patients were evaluated between September 2020 and June 2021. 

### Questionnaires

 A questionnaire with closed questions about sociodemographic data and systemic and oral health was employed. Also, all patients answered the Oral Health Impact Profile (OHIP-14) questionnaire that assesses oral health-related quality of life. 

 The individuals who agreed to participate in the present study were requested to complete a self-assessment questionnaire structured by the authors according to the investigative objectives, containing questions about socioeconomic and demographic aspects, behavioral factors, general and oral health conditions, and items of the OHIP-14 quality of life index. The data were collected in a questionnaire-interview format, where evaluators asked the questions and patients undergoing renal replacement therapy responded according to the given alternatives. An informed consent form (ICF) was also provided, describing the significance of consent for participating in the present study. 

### Sample

 We selected volunteers with CKD (CGA group) undergoing hemodialysis at the HSVP in Passo Fundo, Rio Grande do Sul (n = 116 individuals) to establish the case-cohort for this study. For comparative purposes, a CGO group was recruited with a similar profile regarding sex and age attributes and was comprised of patients of the Faculdade de Odontologia of the Universidade de Passo Fundo, Brazil (n = 124 individuals). 

### Inclusion criteria

 This study included participants who met the following selection criteria:

- CGA group: Individuals who had been undergoing hemodialysis at HSVP, Brazil for at least 1 year, were ≥ 18 years of age, and consented to participate by signing the ICF.
- CGO group: Individuals receiving dental treatment at the Faculdade de Odontologia, Universidade de Passo Fundo, Brazil, who were ≥ 18 years old and agreed to participate by signing the ICF.

### Exclusion criteria

 This study excluded participants who did not meet the following criteria:

- Individuals under the age of 18 because obtaining informed consent from minors is not feasible. Additionally, those with special needs were excluded to ensure that all participants could fully understand and engage with the research requirements. Lastly, individuals who did not provide informed consent to participate were also excluded, in accordance with ethical considerations emphasizing the importance of voluntary participation.

### Pilot study

 A pilot study was performed at the Faculdade de Odontologia of the Universidade de Passo Fundo, Brazil, employing other patients who did participate in this study. The pilot study was necessary to adjust the data collection instruments and identify the most suitable approach method for the analyzed population. 

 The research team (two examiners) was trained and calibrated at the Universidade de Passo Fundo, Brazil, before starting the study, with a theoretical-practical module carried out in a classroom setting using image projections and organized by the coordinator of this research. Examiner agreement was based on the pilot study, obtaining the Kappa coefficient (0.92). 

 The data were tabulated in Microsoft Excel (Microsoft, Redmond, Washington) and the statistical analysis was carried out using SPSS software, version 20.0, (IBM, Chicago). The Chisquare test (categorical variables) analyzed the quantitative data, and the non-parametric Wilcoxon-Mann-Whitney test analyzed the quantitative variables. The accepted statistical significance level between groups was P < 0.05. 

 The OHIP-14 analyses involved working with continuous variables, comparing the mean values of each domain and the overall score according to the group (control or case) by employing oneway analysis of variance, and using SPSS software. The statistical significance level established for all analyses was 5% (P < 0.05). A linear model was tested to verify the mean of the overall score according to the group. 

## RESULTS

 The present study involved the participation of 240 individuals, comprising the CGO group (Control group; Faculdade de Odontologia, Universidade de Passo Fundo, Brazil; n = 124) and the CGA group (Case group; Hospital São Vicente de Paulo, Passo Fundo, Brazil; n = 116). 

 The participants were divided into five age groups. Among the CGO members, the age group of 20–35 years (31.5%) had the highest number of participants. Among the CGA members, the most prevalent age group was 35–50 years old (36.2%). Regarding sex, the CGO group had 59.7% men and 40.3% women, while the CGA group included 58.6% men and 41.4% women (Table 1). 

**Table 1:** Comparison of systemic diseases in the CGO and CGA groups (Wilcoxon Man-Whitney U test; P value)

**Systemic disease**	**CGO, mean**	**CGA, mean**	**P value**
Hypertension	86.36	157.03	0*
Chronic Kidney Disease	62.5	182.5	0*
Diabetes mellitus	103.15	139.05	0*
Respiratory problems	117.48	123.72	0.266
Depression	113.68	127.79	0.008*
Cancer	117.9	123.38	0.098
Anemia	91.94	151.03	0*
Nutritional problems	109.9	131.83	0*
Cardiovascular disease	111.68	111.68	0.001*
Lupus	118	123.17	0.2
Hepatitis C	120	121.03	0.301
**Total**	**124**	**116**	

* Indicates statistical significance between groups; CGO, control group; CGA, case group.

 As for skin color, white skin was the most frequent in both groups: 78.2% in the CGO and 60.3% in the CGA group. Regarding marital status, the CGO group had 42.7% single participants, and the CGA group had 51.7% married individuals. The responses to the employment query showed that 83.6% of the CGO group were working, while 65.3% of the CGA group had worked previously but were not currently employed. When evaluating the education of participants, the results indicated primarily that 50% of the CGO group had completed high school, while 38.8% of the CGA group had completed primary school. 

 As for the most prevalent systemic diseases in the study cohort, the CGO group had hypertension (16.9%), respiratory problems (12.9%), and diabetes mellitus (8.9%). The CGA group showed a higher prevalence of systemic conditions: hypertension (75.9%), anemia (50.9%), and diabetes mellitus (38.8%) (Tables 2, 3). 

**Table 2:** Description of the self-perception of systemic, oral, and chewing health

**Perception of systemic health**	**CGO, n (%)**	**CGA, n (%)**
*Very good*	20 (16.1%)	5 (4.3%)
*Good*	83 (66.9%)	47 (40.58%)
*Regular*	18 (14.5%)	49 (42.2%)
*Poor*	2 (1.6%)	11 (9.5%)
***Very poor***	**1 (0.8%)**	**4 (3.4%)**
**Total**	**124 (100%)**	**116 (100%)**
**Oral health perception**		
*Very good*	19 (15.3%)	5 (4.3%)
*Good*	57 (46%)	58 (50%)
*Regular*	30 (24.2%)	30 (25.9%)
*Poor*	14 (11.3%)	18 (15.5%)
***Very poor***	**4 (3.2%)**	**5 (4.3%)**
**Total**	**124 (100%)**	**116 (100%)**
**Chewing health perception**		
*Very good*	26 (21%)	21 (18.1%)
*Good*	71 (57.3%)	54 (46.6%)
*Regular*	19 (15.3%)	32 (27.6%)
*Poor*	5 (4%)	7 (6%)
***Very poor***	**3 (2.4%)**	**2(1.7%)**
**Total**	**124 (100%)**	**116 (100%)**

CGO, control group; CGA, case group.

**Table 3:** Assessment of self-perception, oral condition, and dental care

**Oral health conditions**	**CGO, n (%)**	**CGA, n (%)**
**Xerostomia**		
*No*	103 (83.1%)	97 (78.2%)
*Yes*	21 (16.9%)	27 (21.8%)
**Total**	**124 (100%)**	**116 (100%)**
		**100 (100%)**
**Last visit to the dentist**		
*Never*	2 (1.6%)	39 (33.6%)
*< 6 months*	81 (65.3%)	21 (18.1%)
*6 months–1 year*	21 (16.9%)	18 (15.5%)
*1–2 years*	10 (8.1%)	17 (14.7%)
*2–5 years*	9 (7.3%)	9 (7.8%)
*5–20 years*	1 (0.8%)	12 (9.6%)
**Total**	**124 (100%)**	**116 (100%)**
**Last service location**		
*Health Center*	17 (13.7%)	51 (44%)
*Private service*	42 (33.9%)	57 (49.1%)
*Other*	1 (0.8%)	6 (5.3%)
*University*	63 (50.8%)	0 (0%)
*Could not inform*	1 (0.8%)	2 (1.8%)
**Total**	**124 (100%)**	**116 (100%)**
**Reason for the last visit**		
*Routine*	35 (28.2%)	22 (19%)
*Pain*	10 (8.1%)	18 (15.5%)
*Bleeding*	3 (2.4%)	1 (0.9%)
*Caries/rest/fill*	21 (16.9%)	15 (12.9%)
*Root canal treatment*	12 (9.7%)	4 (3.4%)
*Extraction*	13 (10.5%)	28 (24.1%)
*Dentures*	7 (5.6%)	18 (15.5%)
*Other*	23 (18.5%)	10 (8.6%)
**Total**	**124 (100%)**	**116 (100%)**
**Pain when chewing**		
*No*	99 (79.8%)	95 (81.9%)
*Yes*	25 (20.2%)	21 (18.1%)
**Total**	**124 (100%)**	**116 (100%)**
**Difficulty chewing solid food**		
*No*	88 (71%)	76 (65.5%)
*Yes*	36 (29%)	40 (34.5%)
**Total**	**124 (100%)**	**116 (100%)**
**Difficulty swallowing**		
*No*	118 (95.2%)	107 (92.2%)
*Yes*	6 (4.8%)	9 (7.8%)
**Total**	**124 (100%)**	**116 (100%)**
**Pain in the mouth or teeth**		
*No*	101 (81.5%)	92 (79.3%)
*Yes*	23 (18.5%)	24 (20.7%)
**Total**	**124 (100%)**	**116 (100%)**
**Pain intensity**		
*Mild*	15 (12.1%)	12 (10.3%)
*Moderate*	10 (8.1%)	8 (6.9%)
*Severe*	0 (0%)	5 (4.3%)
*None*	99 (79.8%)	91 (78.4%)
**Total**	**124 (100%)**	**116 (100%)**

CGO, control group; CGA, case group.

Table 4 shows that the CGA group members displayed the highest mean value in the functional, physical disability, and social disadvantage domains. A generalized linear model showed that the CGO group tended to have lower mean values in the overall OHIP-14 score compared with the CGA group (β = –0.221 (–0.32–0.12; P < 0.001). 

**Table 4:** Mean and standard deviation (SD) according to the overall score and OHIP-14 domains of the control and case groups of hemodialysis patients (n = 240)

**Group**		**Variables**							
		**Overall score**	**Functional domain**	**Physical pain**	**Psychological discomfort**	**Physical disability**	**Psychological disability**	**Social disability**	**Social disadvantage**
CGO(n = 112)	Mean	5.91	0.52	1.72	1.25	0.53	0.96	0.5	0.4
	SD	7.19	1.18	1.94	1.89	1.3	1.56	1.07	0.95
**CGA(n = 116)**	**Mean**	**6.9**	**0.86**	**1.75**	**1.43**	**0.75**	**1.04**	**0.49**	**0.55**
	**SD**	**8.24**	**1.55**	**1.98**	**1.98**	**1.49**	**1.77**	**0.99**	**1.3**
	P*	**0.324**	0.006*	**0.536**	**0.352**	0.042*	**0.165**	**0.592**	0.031*

CGO, control group; CGA, case group; OHIP-14, Oral Health Impact Profile; SD, standard deviation.

## DISCUSSION

 The present study accepts the proposed hypothesis that patients with CKD undergoing hemodialysis have a lower self-perception of quality of life and oral health compared with control patients. Patients with CKD showed a higher prevalence of systemic diseases, such as arterial hypertension, diabetes mellitus, and anemia. These patients also showed the worse indicators of oral health-related quality of life in the functionality, physical disability, and social disadvantage domains. Applying the OHIP-14 questionnaire as well as a clinical and sociodemographic questionnaire to both groups (case and control) ensured the comparison and confirmation of results. 

 Our present findings revealed that men were more prevalent in the samples (CGO 59.7% and CGA 58.6%), corroborating previous studies that showed male prevalence rates of 58%,^17^ 60.6%,^18^ 56.5%,^19^ and 67%.^20^ Regarding age, our study found a prevalence of the 20-35 years age group in the CGO (31.5%) and of the 35–50 years age group (36.2%) in the CGA group. 

 The data on employment and source of income were compelling. Among the 116 patients with CKD, only 5.2% were currently employed, while 58.6% were retired or had a "household" occupation. These data can be explained on the basis that CKD requires permanent treatment, and adequate adherence to regimens and collaboration are conditions for disease control and therapy success.^21^ People often spend years on hemodialysis and have to visit hospitals or specialized clinics up to three times a week for 2–4 hours per visit.^2,11,22^

 As for the most prevalent systemic diseases in the analyzed CKD sample, arterial hypertension was the most prevalent (75.9%) followed by diabetes mellitus (38.8%). Another study on quality of life and CKD found similar results, where arterial hypertension was the most prevalent condition, followed by diabetes mellitus.^10,23^ Epidemiological studies explain these results by claiming that blood pressure and the usual risk factors for cardiovascular diseases are directly related to target organ damage, including vascular stiffness and poor outcomes in hemodialysis patients.^24^ Another study has reported that renal dysfunction related to arterial hypertension contributes to increasing intraluminal hydrostatic pressure.^25^

 Regarding the relationship between diabetes mellitus and CKD in the Brazilian population, approximately 35.6% patients with CKD have systemic arterial hypertension and diabetes mellitus.^22^ This number is much higher than our prevalence data. When linking renal dysfunction to diabetes, described in the literature as diabetic nephropathy, several factors work together by weakening the glomerular basement membrane, expanding the mesangial matrix, decreasing the number of podocytes, and promoting glomerulosclerosis and tubule-interstitial fibrosis.^25^

 The second most prevalent disease in the CGA group was anemia, occurring in half the patients (50.9%). Anemia is defined as hemoglobin levels < 13 g/dL in men and < 12 g/dL in women. Anemia is a common CKD complication resulting from interferences with erythropoietin production.^26,27^ These results can be explained by changes in renal physiology, considering that the kidneys perform multiple functions that can be didactically characterized as filtration, reabsorption, homeostasis, endocrinological and metabolic function.^16^

 Some diseases showed a statistically significant difference when pairing the case (CGA) and control (CGO) datasets and comparing them for systemic health as follows: systemic arterial hypertension (P = 0), CKD (P = 0), diabetes mellitus (P = 0), depression (P = 0.008), anemia (P = 0), nutritional problems (P = 0), and cardiovascular disease (P = 0.001). These results demonstrate that the CGA group is more likely to develop or already have systemic diseases. 

 Oral cavity conditions also affect the quality of life. Thus, the impact of oral health has been studied extensively and has gained importance in the literature.^26-28^ When left untreated and neglected by patients, worsening is frequently observed.^29^ Besides local problems, oral conditions can also work as modifying factors for systemic conditions such as diabetes mellitus, respiratory diseases, and CKD.^30^

 In this context, a comparison between the control and case (CGA; having CKD) groups based on the OHIP-14 score and its internal domains showed a statistically significant difference in the CGA group for functionality (P < 0.006), physical disability (P < 0.042), and social disadvantage (P < 0.031) domains. Overall, the patient group without CKD (CGO group) exhibited a better quality of life compared with those affected by CKD, particularly in the assessed domains.^31-33^ A study performed in Iran with patients undergoing dialysis found a negative impact of oral conditions (assessed with the OHIP-14 questionnaire) on the quality of life of these patients.^34^

 The present study has some limitations. The primary limitation concerns the non-feasibility of performing clinical examinations of the study participants due to the COVID-19 pandemic-related restrictions. Thus, important data such as the Decayed, Missing, and Filled Teeth index, gingival bleeding index, probing depth, radiographic examinations, and oral mucosa lesions could not be measured. Other limitations include the non-feasibility of standardizing the hemodialysis time for the CGA group members and the memory bias and subjectivity of questionnaire responses. 

## CONCLUSION

 Patients with CKD (CGA group) exhibited lower rates of self-perception of oral health and quality of life than individuals in the CGO (control) group. 
